# Supplementary material for: Genome Assembly of the Polyclad Flatworm Prostheceraeus crozieri
Source: Genome Biol Evol. 2022 Aug 30;14(9):evac133. doi: 10.1093/gbe/evac133 (PMC9469890; doi:10.1093/gbe/evac133)
Supplement: evac133_Supplementary_Data [file evac133_supplementary_data.zip › Supplementary figures.docx]

# Supplementary figures

**Supplementary Fig. S1. Maximum likelihood tree reconstruction of *P. crozeri* homeodomains.** Eleven major classes of homeobox genes were identified in *P. crozeri*, PROS class not shown in the tree. Annotations were made based on their relationship to *C. elegans*, Amphioxus and *T. castaneum* homeodomains from HomeoDB. LG+G4 model with 1000 ultrafast bootstraps.
