# Supplementary material for: Genome Assembly of the Polyclad Flatworm Prostheceraeus crozieri
Source: Genome Biol Evol. 2022 Aug 30;14(9):evac133. doi: 10.1093/gbe/evac133 (PMC9469890; doi:10.1093/gbe/evac133)
Supplement: evac133_Supplementary_Data [file evac133_supplementary_data.zip › Supplementary tables.docx]

# Supplementary Tables

| **Supplementary table S1.** GenomeScope genome size and heterozygosity estimations at different Kmer sizes | | |
| --- | --- | --- |
| **Kmer size (bp)** | **Estimated genome size (Gb)** | **Heterozygosity (%)** |
| **19** | 1.56 | 0.936 |
| **21** | 1.59 | 0.922 |
| **23** | 1.61 | 0.896 |
| **25** | 1.63 | 0.872 |
| **27** | 1.65 | 0.849 |
| **29** | 1.67 | 0.829 |
| **31** | 1.68 | 0.810 |

| **Supplementary table S2.** Percentage BUSCO scores for assembly stages and gene annotation | | | | | |
| --- | --- | --- | --- | --- | --- |
|  | **Initial** | **Polished** | **Purged dups** | **Soft-masked** | **Gene annotation** |
| **Complete** | 79.2 | 79.8 | 79.0 | 79.9 | 89.7 |
| **Single** | 73.7 | 76.3 | 75.8 | 77.2 | 87.1 |
| **Duplicate** | 5.5 | 3.5 | 3.2 | 2.7 | 2.6 |
| **Fragmented** | 7.3 | 6.3 | 7.7 | 6.4 | 5.2 |
| **Missing** | 13.5 | 13.9 | 13.3 | 13.7 | 5.1 |

| **Supplementary table S3.** Homeobox annotations in *P. crozieri.* | | | | | |
| --- | --- | --- | --- | --- | --- |
| **ID/position** | **class** | **gene** | **scaffold** | **start** | **end** |
| g10609.t1 | ANTP | Nk1 | ConTiG_47751 | 129 | 184 |
| g12529.t1 | ANTP | Nk2.1 | ConTiG_30465 | 281 | 336 |
| g25651.t1 | ANTP | Nk2.2 | ConTiG_17127 | 316 | 371 |
| g28408.t1 | ANTP | Nk3 | ConTiG_38482 | 217 | 272 |
| g9184.t1 | ANTP | Nk4 | ConTiG_5478 | 152 | 207 |
| g28268.t1 | ANTP | Nk5 | ConTiG_46259 | 246 | 301 |
| g1786.t1 | ANTP | Nk6 | ConTiG_16702 | 149 | 204 |
| g14855.t1 | ANTP | Evx | sCAffold_8727 | 38 | 93 |
| g16381.t1 | ANTP | Cdx | **sCAffold_12245** | 207 | 262 |
| g16400.t1 | ANTP | Hhex | **sCAffold_12245** | 157 | 210 |
| g39727.t1 | ANTP | Hox1 | ConTiG_14153 | 396 | 450 |
| g3922.t1 | ANTP | Hox6-8 | ConTiG_15226 | 264 | 319 |
| g20605.t1 | ANTP | Hox9-13/Post2 | ConTiG_16059 | 181 | 236 |
| g27930.t1 | ANTP | Hox9-13/Post2 | **ConTiG_8557** | 230 | 285 |
| g27942.t1 | ANTP | Hox9-13/Post2 | **ConTiG_8557** | 151 | 206 |
| g13086.t1 | ANTP | Dbx | ConTiG_13408 | 114 | 168 |
| g25523.t1 | ANTP | Lbx | ConTiG_11768 | 133 | 188 |
| g26562.t1 | ANTP | Abox | ConTiG_14304 | 142 | 197 |
| g27829.t1 | ANTP | Tlx | ConTiG_13083 | 102 | 157 |
| g31531.t1 | ANTP | Bsx | ConTiG_9168 | 408 | 463 |
| g33155.t1 | ANTP | En | ConTiG_14650 | 1733 | 1788 |
| g36077.t1 | ANTP | Vax | ConTiG_10002 | 46 | 101 |
| g37735.t1 | ANTP | Dlx | ConTiG_12758 | 178 | 233 |
| g39611.t1 | ANTP | Msx | ConTiG_47676 | 146 | 201 |
| g41386.t1 | ANTP | Msxlx | sCAffold_19200 | 69 | 124 |
| g42084.t1 | ANTP | Emx | ConTiG_5319 | 153 | 208 |
| g42174.t1 | ANTP | Barhl | ConTiG_10582 | 147 | 202 |
| g7617.t1 | ANTP | Barhl | ConTiG_12348 | 105 | 160 |
| g8579.t1 | ANTP | Gsx | ConTiG_15153 | 126 | 181 |
| g9753.t1 | CERS | cers | ConTiG_7915 | 478 | 532 |
| g38497.t1 | CUT | Cux | ConTiG_16288 | 1212 | 1266 |
| g6071.t1 | CUT | Dve_HD1 | ConTiG_19186 | 353 | 419 |
| g6071.t1 | CUT | Dve_HD2 | ConTiG_19186 | 673 | 741 |
| g7562.t1 | CUT | Onecut | ConTiG_10770 | 514 | 567 |
| g23759.t1 | HNF | Hmbox | ConTiG_4249 | 465 | 517 |
| g10273.t1 | LIM | Isl | ConTiG_48104 | 251 | 306 |
| g11350.t1 | LIM | lmx | ConTiG_10122 | 161 | 216 |
| g14032.t1 | LIM | Lhx2/9 | ConTiG_48927 | 258 | 313 |
| g18029.t1 | LIM | Lhx2/9 | ConTiG_49068 | 273 | 328 |
| g22391.t1 | LIM | Lmx | ConTiG_48764 | 406 | 461 |
| g31353.t1 | LIM | Isl | ConTiG_15703 | 205 | 260 |
| g3693.t1 | LIM | Lhx1/5 | ConTiG_7273 | 395 | 450 |
| g40515.t1 | LIM | Lhx3/4 | ConTiG_13260 | 163 | 218 |
| g41036.t1 | LIM | Lhx1/5 | ConTiG_7914 | 178 | 233 |
| g42458.t1 | LIM | Isl | ConTiG_40695 | 205 | 260 |
| g8844.t1 | LIM | Lhx6/8 | ConTiG_7739 | 231 | 286 |
| g15787.t1 | POU | Pou4 | ConTiG_46793 | 346 | 401 |
| g19144.t1 | POU | Pou6 | ConTiG_4929 | 399 | 454 |
| g2650.t1 | POU | Pou3 | ConTiG_42272 | 338 | 393 |
| g42885.t1 | POU | Pou4 | ConTiG_14798 | 293 | 348 |
| g10189.t1 | PRD | Gsc | ConTiG_35351 | 174 | 229 |
| g12353.t1 | PRD | Prop | ConTiG_4595 | 62 | 117 |
| g17080.t1 | PRD | Shox | ConTiG_31705 | 49 | 104 |
| g17360.t1 | PRD | Otp | ConTiG_15879 | 159 | 214 |
| g18105.t1 | PRD | Aprd | sCAffold_49225 | 82 | 137 |
| g18174.t1 | PRD | Pitx | ConTiG_48023 | 124 | 179 |
| g1930.t1 | PRD | Otx1 | **ConTiG_35346** | 114 | 168 |
| g1931.t1 | PRD | Otx2 | **ConTiG_35346** | 76 | 131 |
| g19361.t1 | PRD | Rax | ConTiG_36185 | 31 | 86 |
| g23511.t1 | PRD | Phox | ConTiG_7677 | 108 | 163 |
| g24301.t1 | PRD | Hbn | ConTiG_10596 | 99 | 154 |
| g25130.t1 | PRD | Drgx | ConTiG_46379 | 76 | 131 |
| g30294.t1 | PRD | Vsx | ConTiG_7578 | 190 | 245 |
| g31566.t1 | PRD | Arx | ConTiG_9570 | 55 | 110 |
| g37187.t1 | PRD | Alx | ConTiG_46418 | 68 | 123 |
| g37897.t1 | PRD | Uncx | ConTiG_15889 | 94 | 149 |
| g40557.t1 | PRD | pax4/6 | ConTiG_35872 | 308 | 363 |
| g4231.t1 | PRD | Phox | ConTiG_4077 | 177 | 232 |
| g6157.t1 | PRD | Repo | ConTiG_11112 | 53 | 108 |
| g16992.t1 | SINE | Six4/5 | ConTiG_9909 | 157 | 209 |
| g21138.t1 | SINE | Six3/6 | sCAffold_7399 | 133 | 195 |
| g26271.t1 | SINE | Six3/6 | ConTiG_14082 | 262 | 314 |
| g28637.t1 | SINE | Six1/2 | sCAffold_26188 | 215 | 267 |
| g37748.t1 | SINE | Six4/5 | ConTiG_15522 | 225 | 277 |
| g39976.t1 | SINE | Six3/6 | ConTiG_3805 | 142 | 204 |
| g12490.t1 | TALE | Pbx | ConTiG_31424 | 287 | 345 |
| g14405.t1 | TALE | Irx | ConTiG_50425 | 132 | 189 |
| g21160.t1 | TALE | Phnox | ConTiG_23763 | 192 | 248 |
| g22315.t1 | TALE | Tgif | ConTiG_11953 | 383 | 440 |
| g27697.t1 | TALE | Pknox | ConTiG_18384 | 869 | 925 |
| g33729.t1 | TALE | Irx | ConTiG_11509 | 179 | 237 |
| g4187.t1 | TALE | Irx | ConTiG_29112 | 168 | 226 |
| g18876.t1 | ZF | Zfhx_HD3 | ConTiG_13864 | 2065 | 2120 |
| g18876.t1 | ZF | Zfhx_HD4 | ConTiG_13864 | 2313 | 2368 |
| g2175.t1 | ZF | Zeb | ConTiG_46028 | 405 | 460 |
| g27148.t1 | ZF | Zfhx_HD1 | ConTiG_29319 | 1257 | 1315 |
| g27148.t1 | ZF | Zfhx_HD2 | ConTiG_29319 | 1434 | 1484 |
| g27148.t1 | ZF | Zfhx_HD3 | ConTiG_29319 | 1617 | 1672 |
| g11382.t1 | PROS | pros1 | ConTiG_26943 | 794 | 874 |
| g38366.t1 | PROS | pros2 | ConTiG_11599 | 497 | 572 |
| g2705.t1 | OTHER | OTHER | ConTiG_45877 | 165 | 220 |
| g39033.t1 | OTHER | OTHER | ConTiG_11609 | 68 | 121 |
| g438.t1 | OTHER | OTHER | ConTiG_15163 | 474 | 532 |
